# Effect of Ki-67 Expression Levels and Histological Grade on Breast Cancer Early Relapse in Patients with Different Immunohistochemical-based Subtypes

**DOI:** 10.1038/s41598-020-64523-1

**Published:** 2020-05-06

**Authors:** Qin Liang, Ding Ma, Run-Fang Gao, Ke-Da Yu

**Affiliations:** 1 Department of Breast Surgery, Shanxi Provincial People’s Hospital, Shanxi Medical University, Taiyuan, P.R. China; 2 Department of Breast Surgery, Cancer Center and Cancer Institute, Fudan University, Shanghai, P.R. China

**Keywords:** Breast cancer, Risk factors

## Abstract

This retrospective analysis evaluated the interaction between Ki-67 and histological grade and their prognostic role in different breast cancer subtypes. In total, 2,573 breast cancer patients underwent surgery, and their histological grade and Ki-67 values were evaluated by breast pathologists. The median Ki-67 index was 15%, which was used as the cut-off for low/high Ki-67 expression. Recurrence-free survival (RFS) was calculated and compared, and the results indicated that Ki-67 expression was significantly associated with histological grade in all breast cancer patients (*p* < 0.001) and in each immunohistochemical (IHC)-based subtype (*p* < 0.001). Both high Ki-67 expression and grade 3 tumours were independent predictors of inferior RFS in all patients, especially in those with luminal-like tumours (*p* < 0.05). Ki-67 was an independent prognostic factor for RFS in grade 1, 2 patients with luminal-like tumours (adjusted hazard ratio [HR] = 1.92, 95% confidence interval [CI]: 1.22-3.03, *p* = 0.005), but not in the other subtypes. Similarly, histological grade predicted shorter RFS in patients with low Ki-67 expression who had luminal-like tumours (adjusted HR = 2.12, 95% CI: 1.13-3.99, *p* = 0.02) but not in the other subtypes. Conversely, Ki-67 showed no prognostic value for patients with grade 3 tumours and vice versa.

## Introduction

The American Cancer Society estimates that the most common cancer in females is breast cancer, which accounted for 30% of all new cancer diagnoses in females in the United States in 2018^1^. Since breast cancer is a heterogeneous disease, its adequate evaluation and classification into subtypes based on molecular testing is recommended to predict its prognosis and facilitate treatment decisions. However, the elevated cost and inadequate access to molecular–genetic tests in clinical practice limits its use; therefore, using immunohistochemical (IHC) markers to define breast cancer subtypes is more common^2^. According to previous studies, breast cancer subtypes have an impact on the prognosis of the disease and are associated with the response to endocrine therapy and chemotherapy. Meanwhile, it has been proven that anti-HER2 therapy increases the survival of patients with HER2-positive breast cancer^3^. There are significant differences between the different subtypes of breast cancer.

Tumour proliferation is one of the most important prognostic factors. Ki-67 is a nuclear protein that is present during the late G1, S, G2 and M phases of the cell cycle, reflecting the proportion of cell proliferation^4^. Ki-67 expression is also used for subdividing luminal-like breast cancers into luminal A and luminal B groups^5^. It has also been demonstrated that high Ki-67 expression is associated with a higher risk of relapse and worse survival in early breast cancer patients^6^.

Histological grade is an important determinant of breast cancer prognostication and could be incorporated in staging systems and in algorithms to choose the most appropriate treatment for patients with breast cancer^7^. Histological grade is evaluated by breast pathologists according to the Nottingham modification of the Scarff-Bloom-Richardson (SBR) grading system. The tumour grade is determined by assessing morphological features (tubule formation, nuclear pleomorphism, and mitotic count). Therefore, the tumoral grade may be indirectly related to Ki-67 expression based on the mitotic count. However, few reports have described the correlation between Ki-67 and histological grade and how these two predictive factors are associated with the outcome of patients with different IHC-based subtypes of breast cancer.

In the present study, 2,573 breast cancer patients from a single institute were included to evaluate the interaction between Ki-67 expression levels and histological grade and their prognostic role in different IHC-based breast cancer subtypes.

## Results

### The correlation between Ki-67 and histological grade

As shown in Table 1, Pearson’s χ^2^ test and Student’s *t*-test indicated that Ki-67 expression levels were significantly associated with histological grade in all patients (*p* < 0.001) and in each IHC-based different subtype (*p* < 0.001).

**Table 1:** Correlation of Ki-67 and tumoral grade in breast cancer patients among IHC-based subtypes.

	Ki-67 (continuous)		Ki-67 (categorical)^*^		
	Median (IQR)	*P*	Low	High	*P*
All patients		< 0.001			< 0.001
Grade 1, 2	0.1 (0.05–0.2)		966	743	
Grade 3	0.3 (0.15–0.5)		204	660	
Luminal-like		< 0.001			< 0.001
Grade 1, 2	0.1 (0.05–0.2)		803	430	
Grade 3	0.2 (0.1–0.4)		115	246	
HER2-positive		< 0.001			< 0.001
Grade 1, 2	0.2 (0.1–0.3)		130	256	
Grade 3	0.3 (0.15–0.4)		55	252	
TNBC		< 0.001			< 0.001
Grade 1, 2	0.2 (0.1–0.45)		33	57	
Grade 3	0.4 (0.2–0.65)		34	162	

Abbreviations: IHC-based, immunohistochemical-based; HER2, human epidermal growth factor receptor 2; TNBC, triple-negative breast cancer; IQR, interquartile rage.

* Ki-67 median = 15%, Ki-67 Low <15%, Ki-67 High ≥ 15%.

In addition, the analysis results showed that (Supplementary Fig. S1) to some extent, Ki-67 can predict histological grade, with an area under the curve (AUC) of 0.73 (95% CI: 0.71-0.75) in all patients. Similar results were observed when restricted to certain subtypes (AUC in luminal-like tumours: 0.73, 95% CI: 0.70-0.76; AUC in HER2-positive tumours: 0.63, 95% CI: 0.59-0.67; AUC in TNBC tumours: 0.66, 95% CI: 0.59-0.73).

### Univariable and multivariable analysis of Ki-67 and histological grade on RFS

We studied the association of Ki-67 expression levels and histological grade with RFS (Table 2). In the univariable analysis, both the Ki-67 expression level and histological grade had significant associations with RFS in luminal-like patients (*p* < 0.001) but not in HER2-positive (*p* > 0.05) or TNBC (*p* > 0.05) patients. The multivariable analysis by Cox proportional hazard models adjusting for tumour stage, lymph stage, LVI and IHC-based subtypes confirmed the findings of the univariate analysis. Meanwhile, we divided HER2-positive patients into a luminal HER2-positive subgroup (hormone receptor-positive and HER2-positive) and non-luminal HER2-positive subgroup (hormone receptor-negative and HER2-positive), and conducted survival analyses in the two subgroups. We found that both the univariable and multivariable analyses showed that the Ki-67 expression level and histological grade had no significant associations with RFS in the two subgroups (Supplementary Table S2).

**Table 2:** Univariable and multivariable analyses of Ki-67 and grade on RFS in breast cancer patients among IHC-based subtypes.

	All subtypes			Luminal-like			HER2-positive			TNBC		
	HR	95% Cl	P	HR	95% Cl	P	HR	95% Cl	P	HR	95% Cl	P
**Univariable**												
Ki-67 (categorical)*												
Low (reference)												
High	1.86	1.43–2.42	<0.001	1.97	1.39–2.80	<0.001	1.26	0.76–2.10	0.373	1.38	0.64–3.00	0.413
**Grade**												
1, 2 (reference)												
3	1.77	1.38–2.28	<0.001	2.15	1.50–3.06	<0.001	0.99	0.63–1.54	0.951	1.51	0.74–3.09	0.255
Multivariable**												
Ki-67 (categorical)*												
Low (reference)												
High	1.6	1.21–2.12	0.001	1.88	1.31–2.69	0.001	1.12	0.67–1.87	0.667	1.62	0.73–3.58	0.234
**Grade**												
1, 2 (reference)												
3	1.38	1.05–1.80	0.021	1.97	1.36–2.85	<0.001	0.8	0.51–1.27	0.35	1.31	0.63–2.71	0.466

Abbreviations: RFS, recurrence-free survival; IHC-based, immunohistochemical-based; HER2, human epidermal growth factor receptor 2; TNBC, triple-negative breast cancer; HR, hazard ratio; CI, confidence interval.

* Ki-67 median = 15%, Ki-67 Low <15%, Ki-67 High ≥ 15%.

** Cox proportional hazard models adjusting for tumour stage, lymph stage, lymphatic vessel invasion (LVI) and IHC-based subtype as covariates.

When we treated Ki-67 as a continuous variable rather than as a categorical parameter, the results were similar to the above findings (Supplementary Table S1).

### Interaction between Ki-67 and histological grade in breast cancer patients within IHC-based subtypes

Subsequently, we investigated whether grade and Ki-67 level had complementary effects (Table 3). Univariable analysis showed that in grade 1, 2 breast cancer patients, Ki-67 expression levels had a significant association with RFS in all patients (HR = 2.17, 95% CI: 1.53-3.06, *p* < 0.001) and in luminal-like patients (HR = 2.04, 95% CI: 1.31-3.30, *p* = 0.002), but not in HER2-positive (HR = 1.52, 95% CI: 0.80-2.91, *p* = 0.204) and TNBC (HR = 2.82, 95% CI: 0.59-13.34, *p* = 0.192) patients. Although a higher HR was obtained for TNBC, the evidence for the prognostic importance of Ki-67 in this subgroup was weaker (due to a small number of patients in this subgroup [90 cases]). However, in grade 3 breast cancer patients, Ki-67 expression levels had no significant association with RFS in all patients or in any of the IHC-based subtypes (*p* > 0.05). Further multivariable analysis demonstrated that in grade 1, 2 breast cancer patients, the Ki-67 expression level was a significant independent factor for RFS in all patients (HR = 1.87, 95% CI: 1.30-2.68, *p* = 0.001) and those with luminal-like breast cancer (HR = 1.92, 95% CI: 1.22-3.03, *p* = 0.005) and TNBC (HR = 31, 95% CI: 3.33-288.63, *p* = 0.003). However, because of the low number of cases in this subgroup of TNBC (90 cases), although there was a significant association of Ki-67 with RFS, the high HR was also unbelievable. In grade 3 breast cancer patients, this association did not reach the statistical threshold in all patients or in any of the IHC-based subtypes (*p* > 0.05).

**Table 3:** Univariable and multivariable analyses of RFS in breast cancer patients among different IHC-based subtypes according to the Ki-67 levels of breast cancer patients with different grades.

	All subtypes			Luminal-like			HER2-positive			TNBC		
	HR	95% Cl	P	HR	95% Cl	P	HR	95% Cl	P	HR	95% Cl	P
**Univariable**												
Grade 1, 2												
Ki-67 (categorical)*												
Low (reference)												
High	2.17	1.53 to 3.06	<0.001	2.04	1.31 to 3.20	0.002	1.52	0.80 to 2.91	0.204	2.82	0.59 to 13.34	0.192
Grade 3												
Ki-67 (categorical)*												
Low (reference)												
High	1.03	0.67 to 1.57	0.905	1.12	0.62 to 2.04	0.7	0.951	0.41 to 2.19	0.907	0.89	0.37 to 2.18	0.804
Multivariable**												
Grade 1, 2												
Ki-67 (categorical)*												
Low (reference)												
High	1.87	1.30 to 2.68	0.001	1.92	1.22 to 3.03	0.005	1.46	0.76 to 2.81	0.253	31	3.33 to 288.63	0.003
Grade 3												
Ki-67 (categorical)*												
Low (reference)												
High	1.04	0.67 to 1.62	0.86	1.12	0.59 to 2.10	0.737	0.68	0.29 to 1.60	0.377	0.98	0.40 to 2.42	0.966

Abbreviations: RFS, recurrence-free survival; IHC-based, immunohistochemical-based; HER2, human epidermal growth factor receptor 2; TNBC, triple-negative breast cancer; HR, hazard ratio; CI, confidence interval.

* Ki-67 median = 15%, Ki-67 Low <15%, Ki-67 High ≥ 15%.

** Cox proportional hazard models adjusting for tumour stage, lymph stage, lymphatic vessel invasion (LVI) and IHC-based subtype as covariates.

Similarly, we also analysed the association between histological grade and RFS in patients with different Ki-67 expression levels (Table 4). In “low Ki-67” patients, both the univariable and multivariable analyses showed that histological grade was significantly associated with RFS in all patients (univariable analysis: HR = 2.42, 95% CI: 1.54 to 3.79, *p* < 0.001; multivariable analysis: HR = 1.92, 95% CI: 1.81 to 3.11, *p* = 0.008) and luminal-like patients (univariable analysis: HR = 2.56, 95% CI: 1.43 to 4.56, *p* = 0.001; multivariable analysis: HR = 2.12, 95% CI: 1.13 to 3.99, *p* = 0.02), but not in HER2-positive (univariable analysis: HR = 1.33, 95% CI: 0.53 to 3.35, *p* = 0.539; multivariable analysis: HR = 1.16, 95% CI: 0.44 to 3.05, *p* = 0.762) and TNBC patients (univariable analysis: HR = 3.34, 95% CI: 0.67 to 16.55, *p* = 0.141; multivariable analysis: HR = 3.33, 95% CI: 0.64 to 17.32, *p* = 0.153). Likewise, a higher HR was obtained for TNBC, but histological grade had no significant association with RFS in this subgroup due to the low number of cases (only 67 cases). In “high Ki-67” patients, histological grade had no significant prognostic value either in all patients or in any specific subtype (*p* > 0.05).

**Table 4:** Univariable and multivariable analyses of RFS in breast cancer patients among different IHC-based subtypes according to the grade of breast cancer in patients with different Ki-67 expression levels.

		All subtypes			Luminal-like			HER2-positive			TNBC		
		HR	95% Cl	P	HR	95% Cl	P	HR	95% Cl	P	HR	95% Cl	P
**Univariable**													
Ki-67 Low^*^													
Grade													
1, 2 (reference)													
3		2.42	1.54 to 3.79	<0.001	2.56	1.43 to 4.56	0.001	1.33	0.53 to 3.35	0.539	3.34	0.67 to 16.55	0.141
Ki-67 High^*^													
Grade													
1, 2 (reference)													
3		1.25	0.92 to 1.70	0.162	1.5	0.94 to 2.40	0.088	0.86	0.52 to 1.44	0.57	1.12	0.50 to 2.47	0.789
Multivariable**													
Ki-67 Low^*^													
Grade													
1, 2 (reference)													
3		1.92	1.81 to 3.11	0.008	2.12	1.13 to 3.99	0.02	1.16	0.44 to 3.05	0.762	3.33	0.64 to 17.32	0.153
Ki-67 High^*^													
Grade													
1, 2 (reference)													
3		1.02	0.74 to 1.42	0.891	1.49	0.92 to 2.40	0.104	0.67	0.39 to 1.14	0.136	0.75	0.32 to 1.8	0.525

Abbreviations: RFS, recurrence-free survival; IHC-based, immunohistochemical-based; HER2, human epidermal growth factor receptor 2; TNBC, triple-negative breast cancer; HR, hazard ratio; CI, confidence interval.

*Ki-67 median = 15%, Ki-67 Low <15%, Ki-67 High ≥ 15%.

** Cox proportional hazard models adjusting for tumour stage, lymph stage, lymphatic vessel invasion (LVI) and IHC-based subtype as covariates.

Moreover, we divided HER2-positive patients into a luminal HER2-positive subgroup (hormone receptor-positive and HER2-positive) and non-luminal HER2-positive subgroup (hormone receptor-negative and HER2-positive), and also studied whether grade and Ki-67 level had complementary effects in these two subgroups (Supplementary Tables S3 and S4). The results were similar to those observed in the HER2-positive subgroup, i.e., regardless of the Ki-67 expression level or grade, this association did not reach the statistical threshold in either of the two subgroups (*p* > 0.05).

Combined Ki-67 and histological grade for breast cancer patients within IHC-based subtypes

**Figure 1:**
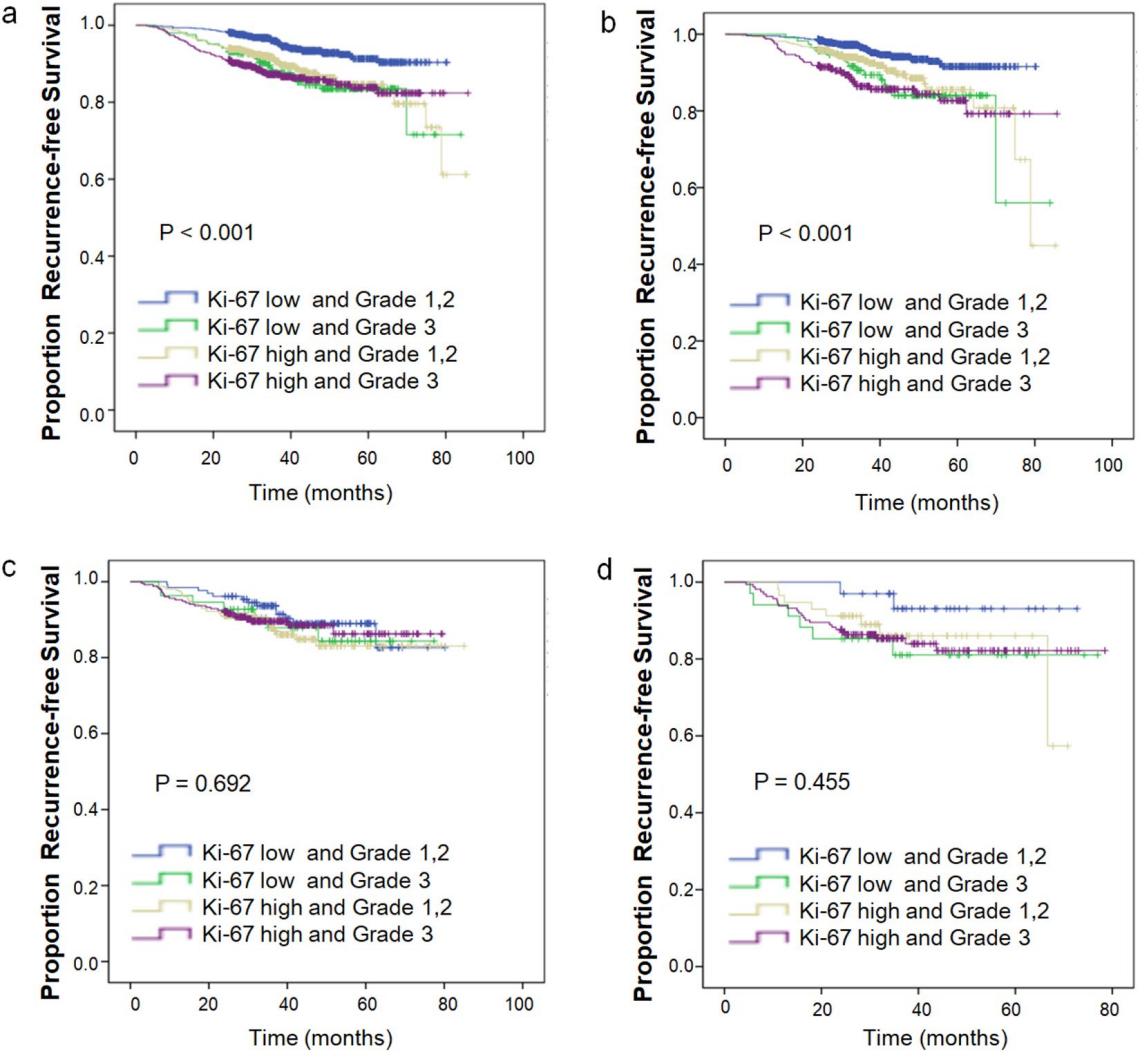
Kaplan-Meier curves of recurrence-free survival according to the different grades and different Ki-67 expression levels of breast cancer patients within different IHC-based subtypes. (**a**) All subtypes; (**b**) luminal-like; (**c**) HER2-positive; and (**d**) TNBC. Abbreviations: IHC-based, immunohistochemical-based; HER2, human epidermal growth factor receptor 2; TNBC, triple-negative breast cancer. NOTE. Ki-67 median = 15%, Ki-67 Low <15%, Ki-67 High ≥ 15%.

The 2,573 breast cancer patients were divided into four groups according to different histological grades and different Ki-67 expression levels: “low Ki-67” and grade 1, 2, “low Ki-67” and grade 3, “high Ki-67” and grade 1, 2, and “high Ki-67” and grade 3. As shown in Fig. 1, the Kaplan-Meier method indicated that for patients in all subtypes and luminal-like patients, “low Ki-67” and grade 1, 2 patients showed significantly higher RFS rates than those in the other three subgroups (*p* < 0.001), while the other three subgroups showed similar RFS rates. However, in patients with HER2-positive and TNBC tumours, the four subgroups had no significant association with RFS (*p* > 0.05). The results indicated that in the HER2-positive and TNBC subgroups, the evidence for the prognostic importance of Ki-67 and grade was weaker, even in the “low Ki-67” and grade 1, 2 patients. However, it should be noted that in the TNBC subgroup, the number of “low Ki-67” and grade 1, 2 patients was very small, only 33 cases, which may affect the analysis results.

## Discussion

The current study indicated that “high Ki-67” was associated with inferior RFS, which was consistent with the findings of previous studies^8,9^. In addition, a meta-analysis including 18 studies also indicated a significant association between Ki-67 and prognosis^10^. The results of previous studies were consistent with those of our study, which detected a statistically significant association between the expression level of Ki-67 and RFS in all patients and in luminal-like patients. However, the present study indicated no significant association between Ki-67 expression levels and RFS in HER2-positive patients and TNBC patients. We considered that the low cut-off point (15% in the present study) to differentiate “high Ki-67” and “low Ki-67” might contribute to the nonsignificance. In the St. Gallen Consensus on the primary therapy of early breast cancer 2011, the cut-off point was set at 14% to distinguish “high Ki-67” from “low Ki-67” to differentiate luminal A and luminal B tumours^11^. HER2-positive breast cancer and TNBC are considered to show more aggressive clinical characteristics and poorer prognosis, showing higher Ki-67 expression levels than luminal-like breast cancer^12,13^. Thus, the cut-off point of Ki-67 might be set higher for HER2-positive and TNBC patients. More prospective studies should be conducted to explore the best cut-off point for the Ki-67 expression level as a predictive factor for RFS in the different clinicopathological subtypes of breast cancer^8,14,15^.

Our research also demonstrated that histological grade was a significant independent factor for RFS in all patients (*p* < 0.001) and luminal-like patients (*p* = 0.021). No significant associations were found between histological grade and RFS in HER2-positive (*p* > 0.05) and TNBC (*p* > 0.05) patients, which was consistent with the findings of previous studies^7^. In addition, a previous study that assessed 1,831 patients also indicated that histological grade showed a very strong correlation with prognosis: patients with a low histological grade had significantly better survival outcomes than those with a high histological grade (*p* < 0.0001)^16^.

The present study also indicated that the trend of association between Ki-67 and RFS was parallel to the trend of association between histological grade and RFS in all patients and those within the different IHC-based subtypes. A positive correlation between Ki-67 and histological grade in breast cancer patients within the different IHC-based subtypes was observed. Interestingly, we found that these two factors can be used together to more accurately predict the prognosis of patients. In grade 1, 2 patients, the association between Ki-67 and RFS was significant in all patients and luminal-like patients. The Ki-67 expression level was a significant independent factor for RFS in patients with grade 1, 2 TNBC, but TNBC only accounted for 11.1% of all breast cancer patients in this study. Interestingly, similar to the above conclusion, the histological grade in all patients and luminal-like patients can help to further divide the prognosis of patients with “low Ki-67”. However, in patients with HER2-positive breast cancer and TNBC, histological grade had no significant prognostic value in patients with either “high Ki-67” or “low Ki-67”. Furthermore, we divided the HER2-positive patients into a luminal HER2-positive subgroup (hormone receptor-positive and HER2-positive) and non-luminal HER2-positive subgroup (hormone receptor-negative and HER2-positive), and investigated the same parameters. The results were similar to those observed in the total HER2-positive subgroup. In other words, our study indicated that in “high Ki-67” patients, histological grade had no significant prognostic value in all breast cancer patients or within any specific subtype. Similarly, in grade 3 patients, Ki-67 also had no significant prognostic value. We considered that patients with “high Ki-67” or grade 3 were associated with decreased RFS and had poorer prognosis, which was consistent with the findings of previous studies^8,9,17,18^. Additionally, HER2-positive breast cancer and TNBC are considered to show more aggressive clinical characteristics and poorer prognosis, resulting in a higher recurrence rate and mortality than luminal-like breast cancer^12,13^. In this study, we also observed that in all patients and luminal-like patients, those with “low Ki-67” and grade 1, 2 had a significant association with better RFS, while in the other three subgroups, patients with different Ki-67 indexes or grades showed similar RFS rates. A recent study detected that all luminal A-type cases and most grade 1 luminal B (HER2-positive)-type cases have low proliferative activity (low Ki-67), whereas histological grade is not informative enough to estimate tumour proliferation (Ki-67 expression levels) in luminal B (HER2-negative), HER2 and TNBC cases^19^. In addition, a study including 1,560 breast cancer patients observed no association between Ki-67 values and histological grade^20^. More studies are needed to provide further evidence for this correlation.

Some limitations of our study should be acknowledged. First, the study did not make different cut-off points of Ki-67 to explore the association between Ki-67 expression levels and RFS in different clinicopathological and histological grade subtypes. Second, because of the low frequency of TNBC in the whole population, the number of TNBC cases only accounted for 11.1% of all the breast cancer patients in this study, while for the TNBC subgroup, the number of “low Ki-67” and grade 1, 2 patients was the smallest in the study, only 33 cases. In addition, the median follow-up time in this study was only 40.6 months. A longer follow-up time is necessary, especially for luminal tumours, where late recurrences are common.

In conclusion, the present study not only showed that Ki-67 and histological grade are important and independent factors for the prognosis of breast cancer, especially for luminal-like cancer, but also, more importantly, that these two factors can be used together to more accurately predict the prognosis of breast cancer patients within different IHC-based subtypes. The association between Ki-67 and RFS was significant in grade 1, 2 patients of all subtypes of breast cancer and luminal-like patients. In “low Ki-67” patients, histological grade had a significant association with RFS in all patients and luminal-like patients. Patients with “low Ki-67” and grade 1, 2 showed significantly higher RFS rates than patients with “low Ki-67” and grade 3, “high Ki-67” and grade 1, 2, and “high Ki-67” and grade 3. The other three subgroups showed similar RFS rates in all patients and in any specific subtype. However, the association between Ki-67 and histological grade is still undefined. Exploring the best cut-off point of Ki-67 for use as a predictive factor for prognosis in breast cancer patients within different IHC-based subtypes is also essential.

## Methods

### Patients

The studied patients were from the Breast Malignancy Database established by the Department of Breast Surgery, Fudan University Shanghai Cancer Center. All patients provided informed consent for their information to be stored in the hospital database and used for research. The information in this database had been reported previously^21^. The present study was approved by the Ethical Committee of Fudan University Shanghai Cancer Center and Institutional Review Boards for clinical investigation. All of the methods were performed in accordance with the Declaration of Helsinki and the relevant guidelines.

In this study, we selected 2,591 consecutive breast cancer patients who were diagnosed with invasive ductal carcinoma (IDC) of the breast and were treated with surgery from Nov 2007 to Dec 2012. All included breast cancer patients met the following inclusion criteria: 1) sufficient information was available to reflect the pathology and follow-up of breast cancer; 2) a tissue sample for IHC was available; and 3) adjuvant systemic chemotherapy, radiotherapy and/or hormone therapy were administered as clinically indicated in accordance with standard practice during the study period. The median time of follow-up was 40.6 months (ranging from 7.9 to 86.7 months). Eighteen subjects were lost to follow-up. Finally, 2,573 patients were included in the present study. Recurrence-free survival (RFS) was the evaluated endpoint. In our study, RFS was defined as the time from when surgery performed to the first recurrence/metastasis of disease or the diagnosis of contralateral breast cancer.

### Pathological report

We determined the tumour stage according to the American Joint Committee on Cancer (AJCC) pathologic tumour-node-metastasis (TNM) classification (7^th^ edition)^22^. The pathological reports included information on the tumour size, histological grade, presence or absence of lymph node metastasis and IHC for hormone receptors (oestrogen receptor [ER] and progesterone receptor [PR]), human epidermal growth factor receptor-2 (HER2) and Ki-67. The positive cell rates for the hormone receptors were determined by IHC, and a value of ≥1% was considered positive^23^. Tumours with a HER2 3+ score on IHC were considered HER2-positive. If the HER2 score was 2+, we performed an immunofluorescence *in situ* (FISH) assay to further evaluate the HER2 amplification status. The fraction of proliferating cells (Ki-67-positive) was based on a count of at least 500 tumour cells in the peripheral area, including the hot spot. The cell count for each image was performed manually using ImageJ software (National Institutes of Health, USA). In each case, the Ki-67 values were presented as the percentage of positive cells. The median value for Ki67 was 15% (range: <1% - 98%), which was used to define low or high Ki-67 expression levels: Ki-67 values <15% were defined as “low Ki-67”, whereas values ≥ 15% were defined as “high Ki-67”. At present, the cut-off between “high” and “low” values for Ki-67 varies between laboratories, as well as between times. Initially, a level of <14% was defined as “low” value for Ki-67 in St Gallen consensus in 2011^3^. Subsequently, the majority of the St Gallen panel voted that a threshold of <20% was indicative of “low” Ki-67 status in 2013^24^. However, we could not determine which one, either 14% or 20%, is a better cut-off for Ki-67 yet. In this study, we chose the median value of 15% for Ki-67 as the threshold. We had further conducted analysis according to the cutoff value of 20% for Ki-67, and the results were consistent with the findings in analysis using 15% as cutoff (data not shown). Histological grade was evaluated by breast pathologists according to Elston and Ellis^25^. Well-differentiated was defined as grade 1, moderately differentiated was defined as grade 2, and poorly differentiated was defined as grade 3.

In this study, the 2,573 breast cancer patients were classified into three subtypes according to immunohistochemistry: (1) luminal-like, which included breast cancer patients who were hormone receptor-positive (ER-positive or PR-positive) and HER2-negative; (2) HER2-positive, which included breast cancer patients who were hormone receptor-positive and HER2-positive or those who were hormone receptor-negative and HER2-positive; and (3) triple-negative breast cancer (TNBC), including breast cancer patients who were hormone receptor-negative and HER2-negative. The basic information on the clinical and pathological characteristics of the breast cancer patients enrolled in this study is shown in Table 5.

**Table 5:** Clinical and pathological characteristics of all the patients.

Characteristics	All patients (n = 2573)	%
**Age at diagnosis, y**		
<40	329	12.8
40-60	1779	69.1
>60	465	18.1
**Tumour stage**		
T1	1192	46.3
T2	1275	49.6
T3	53	2.1
Tx	53	2.1
**Lymph stage**		
N0	871	33.9
N1	1177	45.7
N2	301	11.7
N3	224	8.7
**LVI**		
Negative	1515	58.9
Positive	940	36.5
Unknown	118	4.6
**IHC-based subtypes**		
Luminal-like	1594	61.9
HER2-positive	693	26.9
TNBC	286	11.1
**Histological grade**		
Grade 1, 2	1709	66.4
Grade 3	864	33.6
Ki-67*		
Low	1170	45.5
High	1403	54.5
**Recurrence status**		
Yes	248	9.6
No	2325	90.4

Abbreviations: LVI, lymphatic vessel invasion; IHC-based, immunohistochemical-based; HER2, human epidermal growth factor receptor 2; TNBC, triple-negative breast cancer.

*Ki-67 median value = 15%, Ki-67 Low <15%, Ki-67 High ≥ 15%.

### Statistical analysis

All data were analysed using SPSS, version 21.0 software (SPSS Inc., Chicago, IL, USA). In order to compare the categorical variables, we used the Pearson’s χ^2^ test. The Student’s *t*-test was used to compare the continuous variables between two groups. To estimate the differences between the survival curves that were analysed by the Kaplan-Meier method, we used the log-rank test. Cox proportional hazards model was used to perform the multivariable analyses (method: enter) to further identify the variables that were independently associated with RFS. Cox proportional hazard models adjusting for tumour stage, lymph stage, lymphatic vessel invasion (LVI) and IHC-based subtypes regarded as covariates were used to estimate the independent effects of Ki-67 levels or histological grade on the RFS of breast cancer patients. All *p*-values were from two-sided tests, and a *p*-value of less than 0.05 was considered statistically significant.

## Supplementary information

Supplementary information.

## Data Availability

The datasets analysed during the current study are available from the corresponding author on reasonable request.
