# Supplementary material for: Effect of Ki-67 Expression Levels and Histological Grade on Breast Cancer Early Relapse in Patients with Different Immunohistochemical-based Subtypes
Source: Sci Rep. 2020 May 6;10:7648. doi: 10.1038/s41598-020-64523-1 (PMC7203155; doi:10.1038/s41598-020-64523-1)
Supplement: Supplementary file 1 — Supplementary information. [file 41598_2020_64523_MOESM1_ESM.docx]

**Effect of Ki-67 Expression Levels and Histological Grade on Breast Cancer Early Relapse in Patients with Different Immunohistochemical-based Subtypes**

Qin Liang^1^, Ding Ma^2^, Run-Fang Gao^1^, Ke-Da Yu^2*^

^1^Department of Breast Surgery, Shanxi Provincial People's Hospital, Taiyuan, P.R. China

^2^Department of Breast Surgery, Cancer Center and Cancer Institute, Fudan University, Shanghai, P.R. China

***Corresponding author**

Ke-Da Yu, M.D.

Department of Breast Surgery, Cancer Center and Cancer Institute, Fudan University, 399 Ling-Ling Road, Shanghai, 200032, P.R. China

E-mail: yukeda@163.com

Telephone: 18017317597

## Supplementary information

**Supplementary Table S1.** Univariable and multivariable analyses of RFS in breast cancer patients among IHC-based subtypes with Ki-67 as a continuous variable

|  |  | **Ki-67 (continuous) Univariable** | | |  | **Ki-67 (continuous) Multivariable*** | | |
| --- | --- | --- | --- | --- | --- | --- | --- | --- |
|  |  | **HR** | **95% CI** | **P** |  | **HR** | **95% CI** | **P** |
| All subtypes | | 3.38 | 2.07 to 5.52 | ＜0.001 |  | 2.56 | 1.49 to 4.40 | 0.001 |
|  |  |  |  |  |  |  |  |  |
| Luminal-like | | 4.63 | 2.20 to 9.71 | ＜0.001 |  | 4.4 | 2.06 to 9.39 | ＜0.001 |
|  |  |  |  |  |  |  |  |  |
| HER2-positive | | 1.77 | 0.66 to 4.72 | 0.254 |  | 1.49 | 0.53 to 4.23 | 0.453 |
|  |  |  |  |  |  |  |  |  |
| TNBC | | 1.36 | 0.46 to 4.03 | 0.584 |  | 1.9 | 0.58 to 6.15 | 0.287 |
|  |  |  |  |  |  |  |  |  |

Abbreviations: RFS, recurrence-free survival; IHC-based, immunohistochemical-based; HER2, human epidermal growth factor receptor 2; TNBC, triple-negative breast cancer; HR, hazard ratio; CI, confidence interval.

* Cox proportional hazard models adjusting for tumour stage, lymph stage, lymphatic vessel invasion (LVI) and IHC-based subtype as covariates.

**Supplementary Table S2.** Univariable and multivariable analyses of the association of Ki-67 with RFS in breast cancer patients between the luminal HER2-positive and non-luminal HER2-positive subgroups

|  | | **Luminal HER2-positive** | | | |  | **Non-luminal HER2-positive** | | |
| --- | --- | --- | --- | --- | --- | --- | --- | --- | --- |
|  |  | **HR** | **95% Cl** | | **P** |  | **HR** | **95% Cl** | **P** |
| **Univariable** | |  |  | |  |  |  |  |  |
| Ki-67 (categorical)* | |  |  | |  |  |  |  |  |
|  | Low (reference) |  |  |  | |  |  |  |  |
|  | High | 1.18 | 0.58 - 2.38 | 0.651 | |  | 1.25 | 0.60 - 2.63 | 0.552 |
| Grade | |  |  | |  |  |  |  |  |
|  | 1, 2 (reference) |  |  |  | |  |  |  |  |
|  | 3 | 0.81 | 0.41 - 1.60 | 0.534 | |  | 1.03 | 0.56 - 1.90 | 0.925 |
| **Multivariable**** | |  |  | |  |  |  |  |  |
| Ki-67 (categorical)* | |  |  | |  |  |  |  |  |
|  | Low (reference) |  |  |  | |  |  |  |  |
|  | High | 1.20 | 0.59 - 2.45 | 0.622 | |  | 1.00 | 0.46 - 2.15 | 0.991 |
| Grade | |  |  | |  |  |  |  |  |
|  | 1, 2 (reference) |  |  |  | |  |  |  |  |
|  | 3 | 0.67 | 0.34 - 1.35 | 0.261 | |  | 0.85 | 0.44 - 1.64 | 0.632 |

Abbreviations: RFS, recurrence-free survival; HER2, human epidermal growth factor receptor 2; HR, hazard ratio; CI, confidence interval.

* Ki-67 median = 15%, Ki-67 Low < 15%, Ki-67 High ≥ 15%.

** Cox proportional hazard models adjusting for tumour stage, lymph stage, and lymphatic vessel invasion (LVI) as covariates.

**Supplementary Table S3.** Univariable and multivariable analyses of RFS in breast cancer patients between the luminal HER2-positive and non-luminal HER2-positive subgroups according to the Ki-67 levels of breast cancer patients of different grades

|  | | **Luminal HER2-positive** | | | |  | **Non-luminal HER2-positive** | | |  |
| --- | --- | --- | --- | --- | --- | --- | --- | --- | --- | --- |
|  |  | **HR** | **95% Cl** | | **P** |  | **HR** | **95% Cl** | **P** |  |
| **Univariable** | |  |  | |  |  |  |  |  |  |
| Grade 1, 2 | |  |  | |  |  |  |  |  |  |
|  | Ki-67 (categorical)* |  |  |  | |  |  |  |  |  |
|  | Low (reference) |  |  |  |  |  |  |  |  |  |
|  | High | 1.51 | 0.65 to 3.48 | 0.340 | |  | 1.45 | 0.52 to 4.04 | 0.480 |  |
| Grade 3 | |  |  | |  |  |  |  |  |  |
|  | Ki-67 (categorical)* |  |  |  | |  |  |  |  |  |
|  | Low (reference) |  |  |  |  |  |  |  |  |  |
|  | High | 0.75 | 0.20 to 2.81 | 0.674 | |  | 1.09 | 0.37 to 3.24 | 0.878 |  |
| **Multivariable**** | |  |  | |  |  |  |  |  |  |
| Grade 1, 2 | |  |  | |  |  |  |  |  |  |
|  | Ki-67 (categorical)* |  |  |  | |  |  |  |  |  |
|  | Low (reference) |  |  |  |  |  |  |  |  |  |
|  | High | 1.60 | 0.68 to 3.76 | 0.278 | |  | 0.94 | 0.32 to 2.76 | 0.906 |  |
| Grade 3 | |  |  | |  |  |  |  |  |  |
|  | Ki-67 (categorical)* |  |  |  | |  |  |  |  |  |
|  | Low (reference) |  |  |  |  |  |  |  |  |  |
|  | High | 0.69 | 0.17 to 2.86 | 0.610 | |  | 0.72 | 0.24 to 2.21 | 0.567 |  |

Abbreviations: RFS, recurrence-free survival; HER2, human epidermal growth factor receptor 2; HR, hazard ratio; CI, confidence interval.

* Ki-67 median = 15%, Ki-67 Low < 15%, Ki-67 High ≥ 15%.

** Cox proportional hazard models adjusting for tumour stage, lymph stage, and lymphatic vessel invasion (LVI) as covariates.

**Supplementary Table S4.** Univariable and multivariable analyses of RFS in breast cancer patients between the luminal HER2-positive and non-luminal HER2-positive subgroups according to the grade of breast cancer patients with different Ki-67 expression levels

|  | |  | **Luminal HER2-positive** | | |  | **Non-luminal HER2-positive** | | |
| --- | --- | --- | --- | --- | --- | --- | --- | --- | --- |
|  |  |  | **HR** | **95% Cl** | **P** |  | **HR** | **95% Cl** | **P** |
| **Univariable** | |  |  |  |  |  |  |  |  |
| Ki-67 Low^*^ | |  |  |  |  |  |  |  |  |
|  | Grade |  |  |  |  |  |  |  |  |
|  | 1, 2 (reference) |  |  |  |  |  |  |  |  |
|  | 3 |  | 1.27 | 0.34 to 4.78 | 0.723 |  | 1.25 | 0.34 to 4.68 | 0.736 |
| Ki-67 High^*^ | |  |  |  |  |  |  |  |  |
|  | Grade |  |  |  |  |  |  |  |  |
|  | 1, 2 (reference) |  |  |  |  |  |  |  |  |
|  | 3 |  | 0.68 | 0.31 to 1.51 | 0.344 |  | 0.94 | 0.47 to 1.90 | 0.871 |
| **Multivariable**** | |  |  |  |  |  |  |  |  |
| Ki-67 Low^*^ | |  |  |  |  |  |  |  |  |
|  | Grade |  |  |  |  |  |  |  |  |
|  | 1, 2 (reference) |  |  |  |  |  |  |  |  |
|  | 3 |  | 0.91 | 0.22 to 3.88 | 0.902 |  | 1.29 | 0.31 to 5.30 | 0.728 |
| Ki-67 High^*^ | |  |  |  |  |  |  |  |  |
|  | Grade |  |  |  |  |  |  |  |  |
|  | 1, 2 (reference) |  |  |  |  |  |  |  |  |
|  | 3 |  | 0.52 | 0.23 to 1.19 | 0.123 |  | 0.84 | 0.39 to 1.79 | 0.644 |

Abbreviations: RFS, recurrence-free survival; HER2, human epidermal growth factor receptor 2; HR, hazard ratio; CI, confidence interval.

* Ki-67 median = 15%, Ki-67 Low < 15%, Ki-67 High ≥ 15%.

** Cox proportional hazard models adjusting for tumour stage, lymph stage, and lymphatic vessel invasion (LVI) as covariates.


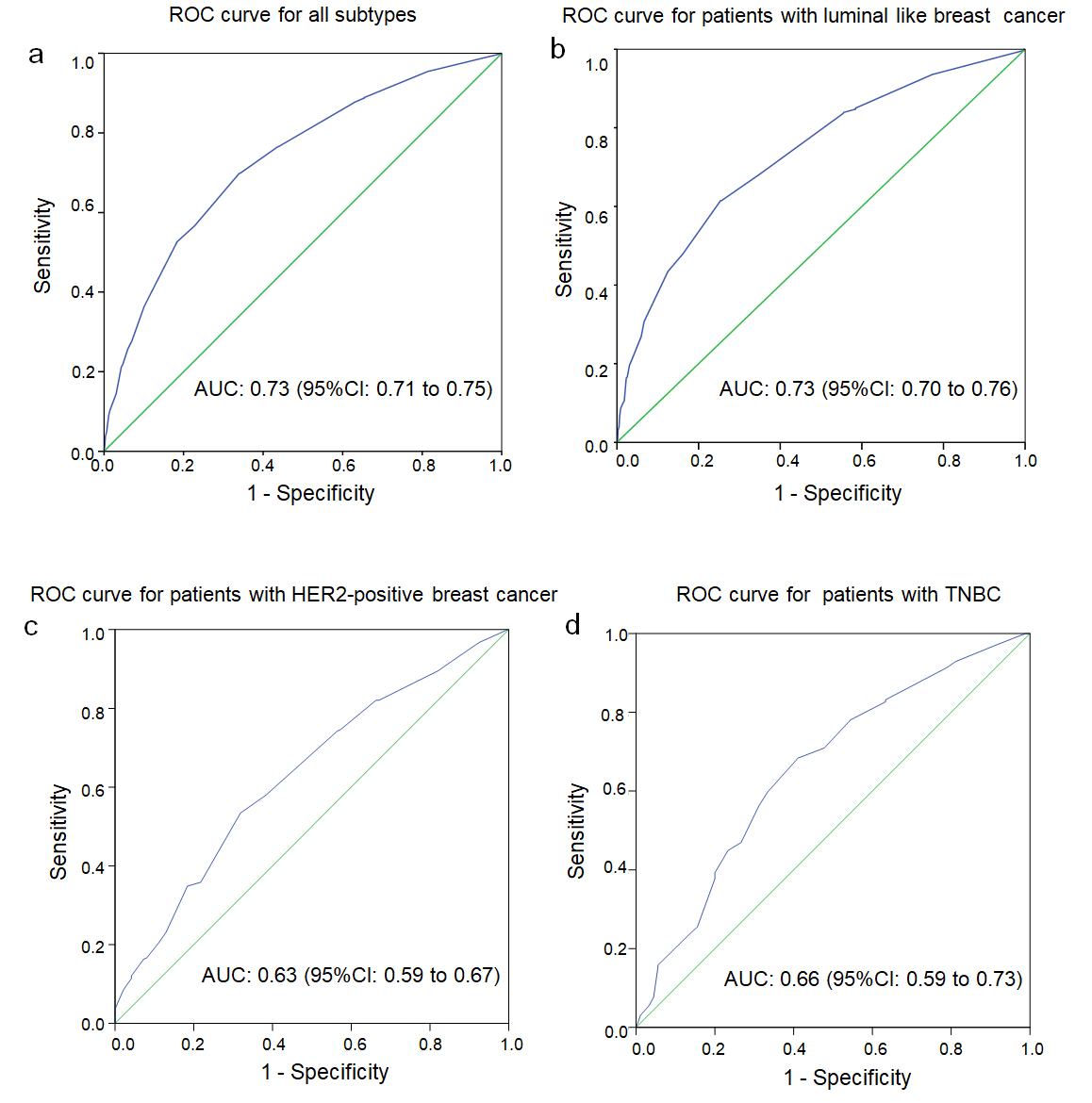


**Supplementary Fig. S1.** ROC curve for predicting histological grade using the Ki-67 expression level of breast cancer patients among IHC-based subtypes. (**a**) All subtypes; (**b**) luminal-like; (**c**) HER2-positive; (**d**) TNBC.

Abbreviations: ROC curve, receiver operating characteristic curve; AUC, area under the curve; IHC-based, immunohistochemical-based; HER2, human epidermal growth factor receptor 2; TNBC, triple-negative breast cancer; CI, confidence interval.
